# The geriatric nutritional risk index independently predicts adverse outcomes in patients with pyogenic liver abscess

**DOI:** 10.1186/s12877-019-1030-5

**Published:** 2019-01-16

**Authors:** Jing Xu, Xinhe Zhou, Chao Zheng

**Affiliations:** Diabetes Center and Department of Endocrinology, The Second Affiliated Hospital and Yuying Children’s Hospital of Wenzhou Medical University, No. 109 West Xueyuan Road, Wenzhou, China

**Keywords:** Geriatric nutritional risk index, Malnutrition, Pyogenic liver abscess, Adverse outcomes, Mortality

## Abstract

**Background:**

Geriatric nutritional risk index (GNRI) is a simple and useful nutritional marker for predictor of adverse outcomes in patients undergoing a variety of conditions. This study explored the relationship between GNRI and adverse outcomes of Pyogenic Liver Abscess (PLA) patients and assessed GNRI predictive value.

**Methods:**

This was one retrospective study involving 240 PLA patients. According to one GNRI cutoff value of 90, the patients were divided into two groups. Besides, demographic, laboratory, adverse outcomes were compared between the two groups. Multivariate logistic regression analyses and receiver-operating characteristic (ROC) curve analysis were performed.

**Results:**

Compared with high GNRI patients, those with low GNRI had a higher risk of mortality (13.4% vs. 2.0%, *P* = 0.002), metastatic infection (17.7% vs. 8.2%, *P* = 0.050), acute hepatic failure (6.8% vs. 1.0%, *P* = 0.036), acute respiratory failure (7.4% vs. 1.0%, *P* = 0.024), upper gastrointestinal (UGI) bleeding (11.9% vs. 2.1%, *P* = 0.006) and empyema (20.1% vs. 10.2%, *P* = 0.047). Multivariate logistic regression analysis demonstrated GNRI (< 90) as one independent factor in death prediction (odds ratio (OR) = 5.36, 95% of confidence interval (CI) = 1.17–24.48), and adverse outcomes (OR = 2.04, 95% CI = 1.05–3.98). GNRI had the largest area under receiver operating characteristic (ROC) curve than albumin, BMI, platelet, prothrombin time and hemoglobin in death prediction (area under ROC curves (AUC) = 0.771, cutoff value = 79.45, *P* < 0.01) and all adverse outcomes (AUC = 0.656, cutoff value = 87.43, *P* < 0.01).

**Conclusions:**

Lower levels of GNRI are an independent risk factor for poor PLA prognosis. Physicians should consider GNRI for PLA outcomes and consider more careful resuscitation and timely and appropriate treatment, especially in those with GNRI< 87.43.

## Background

Pyogenic liver abscess (PLA), although as uncommon disease, can pose a serious threat to individual life, with the incidence rate ranging between 2.3 cases per 10,000 inpatients in North America and 275.4 cases per 100,100 inpatients in Taiwan [1, 2]. Due to improved diagnostic modalities and appropriate treatment, the mortality of PLA has been significantly reduced, ranging between 10 and 40% [3–5]. However, diagnostic and therapeutic issues still exist [6, 7]. In emergency room, it is necessary to identify prognostic markers to offer more timely and aggressive resuscitation to patients, and plan treatment of patients with PLA in the future for good prognosis [7].

It has been well established that the malnutrition is very prevalent among the patients suffering from PLA, showing a close relationship with the elevated risk of cardiovascular and infection-related mortality [8–10]. The Geriatric nutritional risk index (GNRI), an index of malnutrition, has been used as a simple and valuable tool to predict the outcomes calculated from only serum albumin and the ratio between actual and ideal body weight [11]. Over the past years, it has been validated in various studies and its prognostic value has been demonstrated for patients on maintenance haemodialysis (the cutoff value of GNRI was 90) [12], sepsis institutionalized (the cutoff value of GNRI was 87) [13] and also in acutely hospitalized patients (the cutoff value of GNRI was 92) [14].

Although there have been reports concerning a few factors predicting poor PLA prognosis, no prior study is conducted on GNRI utilization to predict the prognosis of patients with PLA. Therefore, the proposed study aims to clarify whether GNRI is associated with adverse outcomes and might be conducive to predict death.

## Methods

### Study design

A retrospective medical record review of all PLA patients admitted to the Second Affiliated Hospital and Yuying Children’s Hospital of Wenzhou Medical University was performed from Jan 2014 to Dec 2016. Through searching institutional database through International Classification of Diseases (9th revision), totally 240 patients with PLA during hospital stay were identified. This study has gained approval from the Institutional Review Board of the hospital. The author waived the requirement for patients’ written informed consent for the anonymity of patients was strictly confidential and the current work was based on observation.

### Study population

The definition of case needed patients to have at least one lesion upon liver imaging (either computed tomography (CT) or ultrasound (US)), along with either complete resolution of radiological abnormalities or one positive blood pus culture following the antimicrobial therapy. In addition, the patients suffering from cirrhosis and amoebic liver abscesses were excluded.

### Data collection and outcome measurements

A retrospective review was conducted on the patients’ clinical records, emphasizing on upon demographic features, such as gender and age; clinical features, such as height, body weight, body temperature and diastolic and systolic blood pressure (BP); Laboratory findings included neutrophil (N) count, white blood cell (WBC) count, hemoglobin (Hb), platelet (PLT), prothrombin time (PT), total cholesterol (TC), activated partial thromboplastin time (APTT), high-density lipoprotein cholesterol (HDL-c), triglyceride (TG), low-density lipoprotein cholesterol albumin (LDL-c), serum BUN, and serum creatinine, etc., all of which were collected from individual medical records on admission according to prespecified definitions. Microorganisms, imaging findings, treatment methods, including antibiotics alone, antibiotics plus percutaneous drainage (PCD), or antibiotics plus surgery, and adverse outcomes, including mortality, empyema, metastatic infection ((defined as distal infection with the same bacterium as culture of PLA); septic shock (defined and treated by Surviving Sepsis Campaign criteria) [15]; acute respiratory failure (defined as requirement of patients for mechanical ventilation); acute hepatic failure (defined as development of severe acute liver injury with encephalopathy and coagulopathy); acute myocardial infarction (one episode of acute myocardial infarction) in hospitalization; acute renal failure (defined as increased serum creatinine> 0.5 mg/dL from baseline) in hospitalization; and upper gastrointestinal (UGI) bleeding (defined as endoscopic evidence of mucosal bleeding related to stress, bright red blood) were recorded for each patient. Besides, the authors observed the mortality for 3 months.

### GNRI calculation

GNRI was calculated from individually obtained height in cm, actual body weight in kg, ideal body weight, and serum albumin levels as following [11]:

GNRI = (1.489*albumin (g/L)) + (41.7*(weight/WL_0_)),

where WL0 denotes the ideal weight, which was calculated by the following formula:

male: WL_0_ = H-100-((H-150)/4).

female: H-100-((H-150)/2.5).

where H denotes the height.

Based on the above values of GNRI, two grades of risk related to nutrition were defined: high risk (< 90) and low risk (≥90) [16].

### Statistical analysis

Continuous data were expressed as means standard deviations or median [interquartile range], and independent t test or Mann-Whitney U test was conducted. Categorical data were expressed as frequencies (%) and were tested using Fisher’s exact or chi-squared test. Univariate logistic regression analysis was adopted for determining probable predictors that showed relationship with the mortality and all adverse outcomes. Each parameter was selected for which *P* value < 0.05 in initial univariate results and age, gender, GNRI and anemia for the multivariate logistic regression analyses model, which was eventually determined with a forward stepwise variable selection procedure. One receiver operating characteristic (ROC) curve was plotted for classifying the mortality and all adverse outcomes of these patients with PLA. The authors analyzed the data utilizing statistical computer programs IBM SPSS Statistics 20.0 (SPSS Inc., USA) and *P* value < 0.05 was deemed significant.

## Results

### The demographic data and underlying disease

240 patients with PLA in total were enrolled. The number of patients with high and low GNRI was 103 (42.9%) and 137 (57.1%), respectively. In comparison with high GNRI patients, the low GNRI patients were older, had lower DBP, and had higher incidences of diabetes, as well as chance of initially presenting with right upper quadrant (RUQ) abdominal pain (*P* = 0.049) (Table 1).

**Table 1:** The comparison of the characteristic and underlying disease between high GNRI and low GNRI patients with pyogenic liver abscess

	Overall (*n* = 240)	High GNRI (≥90, *n* = 103)	Low GNRI (< 90, *n* = 137)	*P*
Age, years	67.6 ± 10.7	62.4 ± 10.4	71.5 ± 9.6	< 0.001^*^
Male	146 (60.8)	71 (68.9)	75 (54.7)	0.032^a^
Height, cm	163.5 ± 7.6	165.4 ± 7.1	162.1 ± 7.7	< 0.001^**^
Weight, Kg	62.5 ± 10.1	67.0 ± 9.6	59.2 ± 9.2	< 0.001^**^
BMI, Kg/m^2^	23.3 ± 2.9	24.4 ± 2.9	22.4 ± 2.7	< 0.001^**^
SBP, mmHg	125 ± 20	124 ± 17	126 ± 22	0.310^*^
DBP, mmHg	71 ± 11	73 ± 12	70 ± 11	0.022^**^
Temperature, ^o^C	39.3 ± 0.8	39.3 ± 0.7	39.3 ± 0.8	0.260^**^
Co-morbidity				
Hypertension	67 (27.9)	26 (25.2)	41 (29.9)	0.469^a^
Diabetes	114 (47.5)	37 (35.9)	77 (56.2)	0.003^a^
Biliary tract disease	53 (22.1)	19 (18.4)	34 (24.8)	0.237^a^
Cancer	19 (7.9)	7 (6.8)	12 (8.8)	0.636^a^
Chronic hepatitis B	6 (2.5)	3 (2.9)	3 (2.2)	1^b^
RUQ pain	75 (31.2)	25 (24.3)	50 (36.5)	0.049^a^
Fever	198 (82.5)	89 (86.4)	109 (79.6)	0.175^a^

Values are expressed as mean ± SD or number (%); *, independent t test; **, Mann-Whitney U test, ^a^chi-squared test, ^b^Fisher’s exact, *BMI* body mass index, *SBP* systolic blood pressure, *DBP* diastolic blood pressure, *RUQ* right upper quadrant

### Laboratory data and liver imaging

*Klebsiella pneumoniae* (KP) was the most common organism that was isolated (74.8%) from both the abscess and the blood cultures of these patients with PLA. In addition, the low GNRI group had significantly higher WBC, N, BUN, PT levels than the high GNRI group. Hb, albumin, uric, TC, HDL-c and LDL-c were remarkably lower in the group with low GNRI that with high GNRI. Regarding imaging parameters for PLA patients, no significant difference was observed between the two groups (Table 2).

**Table 2:** the comparison of the laboratory and liver imaging between high GNRI and low GNRI patients with pyogenic liver abscess

	Overall (*n* = 240)	High GNRI (≥90, *n* = 103)	Low GNRI (< 90, *n* = 137)	P
White blood cell, 10^9^/L	12.2 ± 6.0	11.0 ± 5.6	13.1 ± 6.1	0.005^*^
Neutrophil count, 10^9^/L	10.17 ± 5.89	8.84 ± 5.52	11.17 ± 5.99	0.002^**^
Red blood cell, 10^12^/L	4.0 ± 0.6	4.2 ± 0.6	3.8 ± 0.6	< 0.001^*^
Hemoglobin, g/L	118.9 ± 18.3	126.5 ± 16.6	113.2 ± 17.4	< 0.001^*^
Platelet count, 10^9^/L	222.9 ± 133.9	229.8 ± 123.0	217.8 ± 141.7	0.492^*^
C-reaction protein, mg/L	90.3 ± 68.8	87.2 ± 75.2	93.4 ± 62.4	0.651^**^
Procalcitonin, pg/ml	13.7 ± 23.6	11.9 ± 23.1	14.8 ± 24.0	0.536^**^
Creatinnine, umol/L	72.7 ± 54.1	70.2 ± 22.5	74.6 ± 69.0	0.536^**^
Blood urea nitrogen, mmol/L	5.6 ± 2.9	5.1 ± 1.7	6.0 ± 3.5	0.016^**^
Total bilirubin, mg/L	16.2 ± 14.5	16.5 ± 15.6	15.9 ± 13.6	0.744^**^
ALT, U/L	71 ± 72	69 ± 76	73 ± 69	0.627^**^
AST, U/L	68 ± 86	58 ± 81	76 ± 88	0.126^**^
ALP, IU/dL	199 ± 124	165 ± 104	225 ± 132	< 0.001^**^
Albumin, g/L	30.8 ± 6.7	36.6 ± 5.0	26.5 ± 4.1	< 0.001^*^
Uric acid, umol/L	241 ± 240	258 ± 91	228 ± 96	0.014^**^
TC, mmol/L	3.5 ± 1.0	3.9 ± 1.0	3.3 ± 0.9	< 0.001^*^
TG, mmol/L	1.5 ± 0.9	1.5 ± 0.8	1.6 ± 1.0	0.741^**^
HDL-C, mmol/L	0.6 ± 0.4	0.8 ± 0.4	0.5 ± 0.3	< 0.001^**^
LDL-C, mmol/L	2.1 ± 0.8	2.3 ± 0.8	1.9 ± 0.7	< 0.001^*^
PT, s	14.9 ± 1.6	14.5 ± 1.5	15.1 ± 1.6	0.006^**^
APTT, s	41.8 ± 6.0	41.5 ± 6.0	42.0 ± 5.9	0.486^*^
KP infection	180 (74.8)	79 (76.9)	101 (73.5)	0.818^a^
Abscess features				
Solitary lesion	189 (78.8)	84 (81.6)	105 (76.6)	0.426^a^
Mean size of abscess, cm	6.3 ± 2.8	6.5 ± 3.0	6.2 ± 2.6	0.323^**^

Values are expressed as mean ± SD or number (%); *independent t test; **Mann-Whitney U test, *a* chi-squared test, *ALT* alanine aminotransferase, *AST* aspartate Transaminase, *Alk-P* Alkaline Phosphatase, *TC* total cholesterol, *TG* triglyceride, *HDL-c* high-density lipoprotein cholesterol, low-density lipoprotein cholesterol albumin (LDL-c), *KP Klebsiella pneumoniae*, *PT* prothrombin time, *APTT* activated partial thromboplastin time

### Clinical outcomes

Four (1.7%) patients were treated with antibiotics plus surgery, 110 (45.8%) were treated with antibiotics along, and 126 (52.5%) were treated with antibiotics plus PCD. The low GNRI patients had significantly higher treatment of antibiotics plus PCD than high GNRI patients (*P* = 0.009). Compared with hospitalization expenses of high GNRI group, that of low GNRI group was significantly higher (*P* = 0.035). In comparison with patients with high GNRI (19.4%), those with low GNRI (40.0%) experienced a relative increase of 20.6% in all adverse outcomes’ incidence rate (*P* = 0.001), such as higher mortality rate (13.4% vs. 2.0%), metastatic infection (17.7% vs. 8.2%), acute hepatic failure (6.8% vs. 1.0%), acute respiratory failure (7.4% vs. 1.0%), UGI bleeding (11.9% vs. 2.1%), and empyema (20.1% vs. 10.2%) (Table 3).

**Table 3:** The comparison of the treatment and clinical outcome between high GNRI and low GNRI patients with pyogenic liver abscess

	Overall (*n* = 240)	High GNRI (≥90, *n* = 103)	Low GNRI (< 90, *n* = 137)	*P*
Percutaneous drainage	126 (52.5%)	44 (42.7)	82 (59.9)	0.009^a^
Operation	4 (1.7)	1 (1.0)	3 (2.2)	0.466^b^
Hospital length of stay, days	17.6 ± 9.2	18.3 ± 10.3	17.0 ± 8.2	0.275^**^
Hospitalization expenses, *10^4^ CNY	3.2 ± 2.5	2.9 ± 2.4	3.5 ± 2.5	0.035
Adverse outcomes	75 (31.3)	20 (19.4)	55 (40.0)	0.001^a^
Mortality	21 (8.6)	2 (2.0)	19 (13.4)	0.002^a^
Metastatic infection	33 (13.6)	8 (8.2)	25 (17.7)	0.05^a^
Acute renal failure	9 (3.9)	2 (2,0)	7 (5.2)	0.220^b^
Acute hepatic failure	10 (4.3)	1 (1.0)	9 (6.8)	0.036^b^
Acute respiratory failure	11 (4.7)	1 (1.0)	10 (7.4)	0.024^b^
Acute myocardial infarction	8 (3.4)	3 (3.1)	5 (3.7)	0.783^b^
UGI bleeding	18 (7.8)	2 (2.1)	16 (11.9)	0.006^a^
empyema	38 (15.9)	11 (10.2)	27 (20.1)	0.047^a^
Septic shock	20 (8.2)	7 (6.1)	13 (9.8)	0.346^a^

Values are expressed as mean ± SD or number (%); **Mann-Whitney U test; ^a^chi-squared test, ^b^Fisher’s exact, UGI bleeding, upper gastrointestinal bleeding

### Univariate and multivariate logistic regression analysis

Based on univariate analysis, it can be demonstrated that GNRI< 90, age ≥ 65, anemia, PLT < 125 and PT > 14.8 s were correlated with the mortality; GNRI< 90, age ≥ 65, anemia, PLT < 125 and hypertension were correlated with the adverse outcomes. Through multivariate analysis, it can be found that GNRI< 90, PLT < 125 and PT > 14.8 s were significant predictors of the mortality; GNRI< 90, age ≥ 65 and PLT < 125 were significant predictors of the adverse outcomes in patients with PLA (Table 4 and Table 5).

**Table 4:** Univariate and multivariate logistic regression for risk factors associated with mortality

	Univariate analysis	Multivariate analysis
Variable	Odd ratio (95% CI)	Odd ratio (95% CI)
GNRI< 90	7.45 (1.69–32.91)^*^	5.36 (1.17–24.48)^*^
age ≥ 70 years	4.23 (1.48–12.06)^*^	
Male	0.74 (0.29–1.87)	
Anemia^a^	3.56 (1.31–9.62)^*^	
PLT < 125	3.51 (1.38–8.94)^*^	2.88 (1.03–8.00)^*^
PT > 14.8 s	3.23 (1.19–8.72)^*^	3.37 (1.13–10.11) ^*^
Percutaneous drainage	0.80 (0.32–2.02)	
size> 6 cm	1.92 (0.64–5.75)	
creatinine> 1.3 mg/dL	2.50 (0.65–9.55)	
Diabetes	0.71 (0.28–1.80)	
hypertension	0.84 (0.29–2.43)	

^a^Hemoglobin < 13 g/dL in men, < 12 g/dL in women, *CI* confidence interval

*P < 0.05

**Table 5:** Univariate and multivariate logistic regression for risk factors associated with adverse outcomes

	Univariate analysis	Multivariate analysis
Variable	Odd ratio (95% CI)	Odd ratio (95% CI)
GNRI< 90	2.77 (1.51–5.09)^*^	2.04 (1.05–3.98)^*^
age ≥ 70 years	2.14 (1.22–3.76)^*^	1.95 (1.05–3.64)^*^
Male	0.79 (0.45–1.39)	
Anemia	1.81 (1.03–3.17)^*^	
PLT < 125	3.11 (1.67–5.78)^*^	2.98 (1.53–5.82)^*^
PT > 14.8 s	1.72 (0.99–3.01)	
Percutaneous drainage	0.71 (0.41–1.23)	
size> 6 cm	1.02 (0.48–2.15)	
creatinine> 1.3 mg/dL	1.80 (0.95–3.39)	
Diabetes	1.74 (0.99–3.05)	
hypertension	1.82 (1.00–3.31)^*^	

^a^Hemoglobin < 13 g/dL in men, < 12 g/dL in women, *CI* confidence interval

*P < 0.05

### Prognostic value of GNRI

**Fig. 1:**
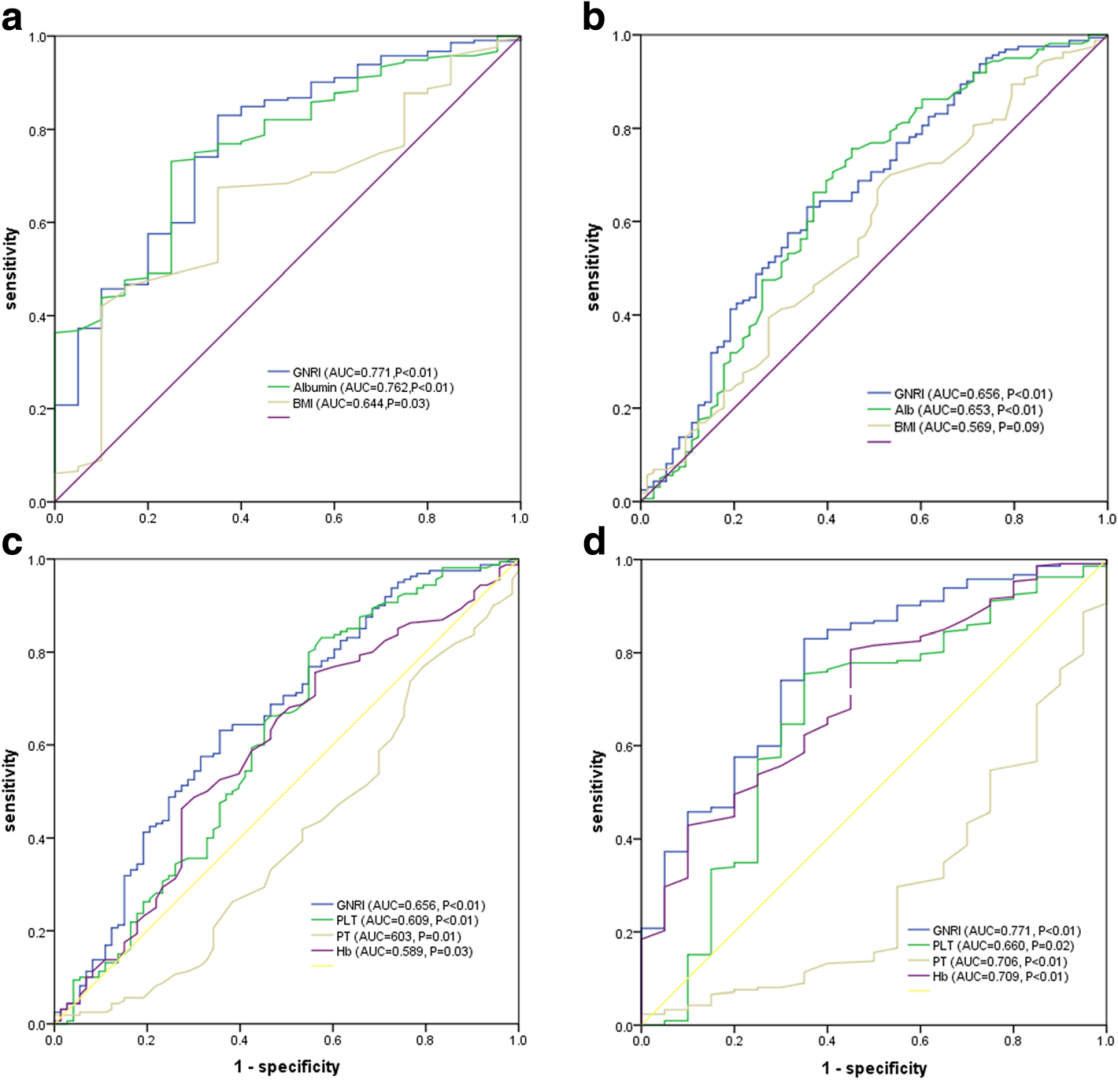
**a** ROC analysis of GNRI, albumin and BMI to mortality among PLA patients. **b** ROC analysis of GNRI, albumin and BMI to adverse outcomes among PLA patients. **c** ROC analysis of GNRI, platelet, prothrombin time and hemoglobin to mortality among PLA patients. **d** ROC analysis of GNRI, platelet, prothrombin time and hemoglobin to adverse outcomes among PLA patients

To compare the predictability of the mortality and all adverse outcomes among PLA patients, ROC curves for GNRI, albumin, BMI, platelet, prothrombin time and hemoglobin were plotted, as shown in Fig. 1a-d. GNRI had the highest area under the ROC curves, with statistical significance (0.771 for mortality and 0.656 for all adverse outcomes). The optimal cutoffs of GNRI for predicted mortality and all adverse outcomes were 79.45 and 87.43, respectively.

## Discussion

Admittedly, the proposed study is the first attempt to assess the relationship between GNRI and PLA prognosis. The following is the summary of the major findings. Firstly, the authors demonstrated that compared with PLA patients with high GNRI, those with low GNRI had worse prognoses (including mortality, metastatic infection, acute hepatic failure, acute respiratory failure, UGI bleeding, and empyema). Secondly, the low GNRI independently predicted adverse outcomes of PLA patients during hospitalization. Thirdly, in comparison with serum albumin, BMI, platelet, prothrombin time and hemoglobin, the GNRI served as a more useful parameter in prediction of the adverse outcomes of PLA patients.

Considering that PLA is usually within one heightened proinflammatory state and does damage to liver anabolism, (which makes its nutritional status significantly worse) [17, 18], malnutrition’s effects may be more amplified. Consistent with this, mean serum albumin in the current study was 30.8 g/L, lower than the normal serum concentration. It has been well established that the malnutrition is very prevalent among the patients suffering from PLA, showing a close relationship with the elevated risk of cardiovascular and infection-related mortality [10]. Hence, nutritional management is greatly important for the patients with PLA. Calculated from both the BMI and serum albumin, geriatric nutritional risk index is a comparatively novel evaluation tool for evaluating the severe or chronic diseases of patients [19, 20]. Previous study has revealed that GNRI is one simple acute-risk assessment tool for the short-run in-hospital mortality rate of sepsis patients [13]. Nevertheless, GNRI clinical utility in predicting mortality related to PLA is still remains unknown, and there is a lack of evidence to show if GNRI has an improvement effect on predictive value for risk evaluation beyond present traditional nutritional markers.

There is another significant finding of the proposed study, that is, adding GNRI into one model that involves potential risk factors is remarkably superior to adding BMI or serum albumin alone into that model in the aspect of the prediction of adverse outcomes and PLA-related death. Serum albumin is considered as a clinical monitoring tool for nutrition assessment, and hypoalbuminemia is strongly correlated with the complications and mortality in the elderly [21, 22]. Both, BMI and serum albumin are linearly associated with reduced risk of infection-related death [23, 24]. Additionally, both the serum albumin and BMI are affected by a few non-nutritional factors, including inflammation, renal dysfunction, and fluid status [25, 26]. Therefore, GNRI predictive value, one dualistic assessment of serum albumin and BMI, can be complementary and enhance the accuracy of diagnosis through handling all parameters’ limitations. Actually, as presented in Fig. 1a, b, GNRI had the largest area under ROC curve compared with BMI and serum albumin, with statistical significance.

The result shows that malnutrition has an independent relationship with the increased hospitalization expense, longer hospital stay, and higher infection risk [8, 9]. The results of our study correspond to those of previous studies concerning PLA of patients with low GNRI, who has higher WBC, N, BUN, PT, hospitalization expense, percutaneous drainage rate and had lower Hb levels than those with high GNRI. Considering the multiple risk indexed associated with adverse outcomes, a lower GNRI indicates that physicians should consider more careful resuscitation and timely and appropriate treatment.

A few previous research has reported other values of prognosis for PLA [6, 7, 27], which included older age, increased serum creatinine and BUN, low hemoglobin and albumin levels, biliary origin’s liver abscess, polymicrobial infection, pleural effusion, concomitant malignancy, and multiple abscesses. Consistently, the present study found that GNRI< 90, PLT < 125, PT > 14.8 s were associated with the mortality of PLA, and that GNRI< 90, age > 65, PLT < 125 were associated with all poor prognoses in PLA. So far, the most suitable biomarker for the prediction of liver abscess has not been created. In the current work, as shown in Fig. 1c and d, GNRI had the largest area under ROC curve compared with platelet, prothrombin time and hemoglobin, with statistical significance. Additionally, GNRI has demonstrated an apparent superiority as one biomarker, which is easy-to-use, economical, readily available, requiring patients’ minimal participation.

Complicated synergistic effects of atherosclerosis, inflammation, and malnutrition may explain the strong relationship between clinical outcomes and malnutrition. Moreover, as nutritional status is related to immunity, patients with poor nutritional status undergoing from infection are more common [28]. The weakened immune system in these malnourished patients may be contributed to explaining the high rate of infection-related complications and mortality.

This study clearly demonstrated that GNRI is predictive of the outcomes of mortality. Existing prognostic models for mortality, such as SAPS 3 and APACHE 2 generally utilize traditional predictive variables, such as admission diagnosis, medical history, physiological variables, and/or medical treatment for mortality prediction [29, 30]. Since our study has shown that nutritional status has independent value of prognosis, the future model should take the nutritional status into consideration when mortality in PLA is prognosticated.

Moreover, it has been observed that from admission to discharge, there are dynamic changes within the serum albumin [22]. Improving the serum albumin from low level to normal level may be conductive to improving mortality. In addition, we suggest the necessity of timely and appropriate nutritional support to improve clinical outcomes, especially to reduce mortality in PLA patients.

It is noteworthy that the present study still has a few limitations. Firstly, as this study was one single-center retrospective study, one selection bias and one problem in results generalization occurred. Secondly, PLA patients’ mortality rate during hospitalization was substantially smaller (21 cases of death). Thirdly, the analyses used one single GNRI measurement at baseline, which may fail to catch the intra-individual variability with time passing and led to patient misclassification in different categories of GNRI levels. Furthermore, one single GNRI measurement at baseline was utilized for conducting the analysis. GNRI predictive value varies with time, providing information in more detail of the causative effects. Nevertheless, no GNRI change with time is reflected in our data. Further research should be conducted for determining the influence of GNRI changes with time upon mortality and all adverse outcomes.

## Conclusions

To conclude, one lower GNRI has a strong relationship with adverse outcomes of PLA patients. The findings of our study indicate that GNRI predictive value for the prediction of adverse outcomes is superior to that of serum albumin. If GNRI is < 87.43 in initial presentation of patients with PLA, physicians are supposed to predict poor PLA prognosis and should consider more careful resuscitation and timely and appropriate treatment.

## Abbreviations

GNRI: Geriatric nutritional risk index
KP: *Klebsiella pneumoniae*
N: Neutrophil count
PCD: Percutaneous drainage
PLA: Pyogenic liver abscess
RUQ: Right upper quadrant
UGI: Upper gastrointestinal
WBC: White blood cell count
